# Characterization of nuclear mitochondrial insertions in the whole genomes of primates

**DOI:** 10.1093/nargab/lqaa089

**Published:** 2020-11-16

**Authors:** Gargi Dayama, Weichen Zhou, Javier Prado-Martinez, Tomas Marques-Bonet, Ryan E Mills

**Affiliations:** Department of Computational Medicine and Bioinformatics, University of Michigan Medical School, Ann Arbor, MI 48109, USA; Department of Computational Medicine and Bioinformatics, University of Michigan Medical School, Ann Arbor, MI 48109, USA; Wellcome Sanger Institute, Hinxton, CB10 ISA, Cambridge, UK; Institute of Evolutionary Biology (UPF-CSIC), PRBB, Dr. Aiguader 88, 08003 Barcelona, Spain; Catalan Institution of Research and Advanced Studies (ICREA), Passeig de Lluís Companys, 23, 08010, Barcelona, Spain; CNAG-CRG, Centre for Genomic Regulation (CRG), Barcelona Institute of Science and Technology (BIST), Baldiri i Reixac 4, 08028 Barcelona, Spain; Institut Català de Paleontologia Miquel Crusafont, Universitat Autònoma de Barcelona, Edifici ICTA-ICP, c/ Columnes s/n, 08193 Cerdanyola del Vallès, Barcelona, Spain; Department of Computational Medicine and Bioinformatics, University of Michigan Medical School, Ann Arbor, MI 48109, USA; Department of Human Genetics, University of Michigan Medical School, Ann Arbor, MI 48109, USA

## Abstract

The transfer and integration of whole and partial mitochondrial genomes into the nuclear genomes of eukaryotes is an ongoing process that has facilitated the transfer of genes and contributed to the evolution of various cellular pathways. Many previous studies have explored the impact of these insertions, referred to as NumtS, but have focused primarily on older events that have become fixed and are therefore present in all individual genomes for a given species. We previously developed an approach to identify novel Numt polymorphisms from next-generation sequence data and applied it to thousands of human genomes. Here, we extend this analysis to 79 individuals of other great ape species including chimpanzee, bonobo, gorilla, orang-utan and also an old world monkey, macaque. We show that recent Numt insertions are prevalent in each species though at different apparent rates, with chimpanzees exhibiting a significant increase in both polymorphic and fixed Numt sequences as compared to other great apes. We further assessed positional effects in each species in terms of evolutionary time and rate of insertion and identified putative hotspots on chromosome 5 for Numt integration, providing insight into both recent polymorphic and older fixed reference NumtS in great apes in comparison to human events.

## INTRODUCTION

Polymorphic Nuclear Mitochondrial Insertions (NumtS) are fragments of mitochondrial DNA (mtDNA) that have been transferred and integrated into the nuclear genome of an organism. NumtS vary in size from smaller fragments to full-length mitochondrial integrations and can share varying degrees of homology with the genome of their parent mitochondria, depending on the age and time of insertion. Once these pieces of mtDNA have inserted themselves, their mutation rate decreases by an order of magnitude to the background rate of the organism's nuclear genome, essentially fossilizing the fragment and providing a snapshot of the ancestral mitochondria from when it was inserted (1–3). This information can be very beneficial in phylogenetic analysis (4,5), and indeed such inserted sequences have been identified in previous studies exploring their prevalence in humans (5–7), chimpanzees (5,8–9) and other organisms (5,10). As with other forms of genetic variation, NumtS can be both fixed within all genomes of a species as well as polymorphic between individuals. While the majority of prior studies have focused on older, fixed insertions, recent efforts have begun to explore the impact of segregating Numt alleles within human populations (2,7).

It has been shown that all parts of the mtDNA are able to transfer into the nuclear genome, though relative rates for individual regions are still unknown (7). Prior studies have reported a deficit of NumtS from the hypervariable (HV) regions of mtDNA, specifically HV2 within the mitochondrial control region (MCR_F_) (1). In particular, a negative correlation was reported between the prevalence of NumtS and the proportion of variability of the site, thus increasing the difficulty of detecting events in the nuclear genome, which exhibits a slower rate of mutation (1). More recent work focusing specifically on Numt polymorphisms, however, reported a slight enrichment of the D-loop for the polymorphic events (7). Likewise, the patterns of insertional preferences within chromosomal DNA have not been well defined. Earlier work reported that younger insertions are more prevalent within intronic regions, while older NumtS tend to be intergenic (11). More recent analyses have suggested that they may insert non-randomly, with a tendency to insert in regions significantly deficit of transposable elements (12,13), while also seeming to have a local affinity toward low GC regions and A+T dinucleotides in humans (7). An abundance of human specific NumtS on chromosome Y was reported in one study, though these appear to have arisen through the duplication of existing NumtS and were likely not driven through new insertions (1). However, others have reported no pattern or specific insertion sites (14).

Previous studies have also found discrepancies in the rate of insertion for NumtS across different species. For example, some groups have observed a consistent rate of insertion throughout the evolution of great apes (1,6,15), whereas others reported variability between species (9,16). Indeed, earlier observations reported a higher rate of insertions in both gorillas (17) and chimpanzees (12) as compared to humans. Additional research has reported a burst of insertion at the divergence of old-world and new-world monkeys (5,15,18–19). Further, an interesting stratification in the evolution of fixed Numt insertions was also reported between primate and non-primate species (5). Overall, there is much ambiguity regarding the interspecies rate of insertion, copy number and length and these discrepancies are likely due in part to the predominant use of older, species-specific NumtS as well as limited sample size in the prior analysis.

Here, we present a holistic analysis of NumtS using both older, fixed insertions as well as new polymorphic events detected by our previously developed methodology (7). We derive the prevalence and insertion rates of NumtS across species and explore positional effects and sequence context. We further *de novo* assemble a subset of Numt insertions and analyze their evolutionary distance from constructed ancestral mtDNA sequences for the various speciation events. This represents the first exploration and assessment of polymorphic events and their significance in the evolution of the primates.

## MATERIALS AND METHODS

### Data acquisition and processing

Whole genome, paired-end sequencing data were obtained in SRA format from the short read archive (https://www.ncbi.nlm.nih.gov/sra) for various non-human primates: chimpanzee—*Pan troglodytes*; bonobo—*Pan panicus*; gorilla— *Gorilla gorilla* and orang-utan—*Pongo abelii* and *Pongo*  *pygmaeus*, were obtained from the Great Ape Genomes Project (20) and rhesus—*Macaca mulatta* data were obtained elsewhere as an outgroup (21). The SRA files (SRP018689, ERP002376) were converted to fastq format using the SRA Toolkit (version 2.3.5-2 released April 2014) and then subsequently aligned to its respective species-specific reference and processed using BWA (22) and GATK (23) to generate alignment files in BAM format. The reference versions used were as follows; for chimpanzee ‘panTro4’, for bonobo ‘panPan1’ (assembled by (24)), for gorilla ‘gorGor4’, for orang-utan ‘ponAbe2’ and for rhesus ‘rheMac3’, and comparisons were made to previously identified NumtS in humans using the GRCh37 version of the human reference genome.

### Numt identification and assembly

To reduce misalignments from variability in mtDNA sequences within individuals of each species, modified references were generated for each sample by concatenating individual mtDNA to its species-specific reference. The sample-specific mtDNA were assembled using mtArchitect, a method previously developed (25) which uses iterative mapping whereby sequence reads initially aligning to the reference mtDNA for each individual were isolated and assembled into a single mtDNA contig. These sample-specific mtDNA sequences were then inserted into each respective reference genomes as an additional contig and sequence reads were realigned and processed as described above. The updated BAM files were used for polymorphic Numt discovery using dinumt (7), which utilizes aberrant/discordant reads (where one read maps to the mtDNA and the other elsewhere on the nuclear genome), read orientation and various other filters to define insertion breakpoints. Fixed NumtS in each species-specific reference genome were reanalyzed using the method previously described by (26) to generate a consistent and updated set across organisms.

A three step process was utilized for the *de novo* assembly of non-reference NumtS detected by our approach: (i) Clustering: the aberrant and soft-clipped read pairs from the flanking region of non-reference Numt insertion sites were extracted and collated from all the samples for which with the insertion was detected. (ii) Assembly: where possible, these polymorphic NumtS were then assembled into contigs using CAP3 (27). (iii) Annotation: the resulting contigs were subsequently aligned against their respective reference mtDNA genome to annotate the specific Numt fragment.

### Orthogonal validation of NumtS using long-read sequences

To assess the accuracy of putative Numt insertion sites obtained from the short-read data sets, we made use of existing long-read whole genome sequences that were available for a subset of the species we interrogated. Long-read sequencing data from Pacific Biosciences (PacBio) Single Molecule, Real Time (SMRT) technology were obtained from NCBI under the project accession numbers PRJNA369439 (chimpanzee, orang-utan) and PRJEB10880 (gorilla) (28,29). We used pbmm2 (https://github.com/PacificBiosciences/pbmm2) to align the PacBio raw reads of three primate genomes to their relative references. We customized an approach for identifying mobile element insertions, PALMER (30) (https://github.com/mills-lab/PALMER), to allow the discovery of NumtS in PacBio aligned data. We required at least four sub-reads at a given locus with NumtS-related sequences and performed additional error correction (sequencing error <1%) using CANU (31) to correct PacBio sub-reads that support the insertions. These were subsequently re-aligned locally to the predicted insertion sites using *pbmm2*, followed by another of PALMER to better define the sequences and integration sites of the detected NumtS. We then examined the intersection between calls from PacBio and standard short reads, allowing an extension of ±500 bp for insertion site position.

### Frequency and characterization of insertion site across all the species

To investigate whether insert-size or coverage had an effect on the number of insertions we observed, we derived and compared the frequency of Numt insertions relative to their respective reference for every non-human primate sample. The defined Numt breakpoints (site of insertion in the genome relative to the reference) were then unified to the human reference (GRCh37) using the lift-over tool and chain files from UCSC Genome Browser (32) to compare the sites of insertion across all primates including humans (7) for both reference and polymorphic events. They were then visualized using a circos (33) plot.

A similar strategy was used to assess the subset of assembled NumtS. Their aligned coordinates were unified to the human reference (GRCh37) as described above and a circos plot was generated to display integration preferences for mtDNA fragments into each species. The human reference mtDNA (GRCh37) was binned into genes and the average rate of insertion for each bin was calculated across all species.

We also utilized these liftover coordinates as unified to the human reference (GRCh37) from all other species to define ancestral NumtS positions. Polymorphic and fixed NumtS from each species were combined to conduct a pairwise assessment of shared events across all species. Ancestral NumtS at each node of divergence were determined by combining all species that diverged at the given node and screening for shared events relative to humans. Furthermore, we also examined shared events between all species relative to each other in a pairwise fashion, omitting bonobo to avoid any bias introduced due to their unusually low number of shared events.

### Characterization and rate of insertions for NumtS

To investigate species-specific differences in insertional characteristics, we compared the fragment length and GC content for all the assembled polymorphic and reference NumtS (extracted from the reference genome of each species). In addition, the rate of insertion was estimated for assembled polymorphic NumtS using the method described previously (7). The ancestral mitochondrial sequences for each primate genus were obtained from ENSEMBL Compara release 77 based on 8 primates EPO (Enredo-Pecan-Ortheus) pipeline (34). A profile of nucleotide changes was generated between these ancestral mtDNA and the consensus mtDNA for each species. The assembled non-reference NumtS were then annotated for these specific positions to calculate an allele ratio. Finally, after post-correction of any mismatches, these allele ratios were normalized by the evolutionary distance since their specific node divergence, thus calculating the approximate age of each assembled insertion.

### Enrichment analysis

We tested for any potential preferential bias of MT genes being inserted as NumtS. To assess this, we performed a permutation analysis by generating 1000 sets of random insertion coordinates based on the list of derived polymorphic and reference Numt fragment sizes. The frequency of complete gene insertions was calculated for all MT genes for each species. As some of the primate species were lacking the MT gene annotation, we defined the genes using human mtDNA reference (GRCh37) for each primate species through the lift-over tool and chain files from UCSC Genome Browser (32). A Z-score was calculated for each gene to determine trends of MT gene enrichment or depletion across all human and non-human primates as follows:

$$\begin{equation*}{\bf Z - score}\,{\rm{ = }}\,\left( {{\rm{x - \mu }}} \right){\rm{/\sigma }}\end{equation*}$$

where x is the true values of total gene insertions, μ is the mean of permutation count and σ is the standard deviation of the permutation count.

### Analysis of putative hotspots of Numt insertion

The entire nuclear genome was divided into 25 Mbp bins and the frequency for Numt insertions was calculated and compared across the homologous positions in each species to determine any hotspots above a permutation threshold that defines 5% FDR (false discovery rate) as follows:

$$\begin{equation*}\sqrt {\mathop \sum \limits_{i\ = \ 1}^n {{\left( {\underline {{x_i}} - {x_i}} \right)}^2}} \end{equation*}$$

Where $\underset{\scriptscriptstyle-}{x}$ is the mean frequency of NumtS across all the bins for a given species *i* and $x$ is the frequency of NumtS in each bin, equated across $n$ number of species. A total of 25 Mbp was determined to be a lower bound for window size given the sparsity of reference and polymorphic NumtS in each species.

### Software availability

The software and analysis pipelines used in this analysis can be found at https://github.com/mills-lab/primate_numt and https://github.com/mills-lab/dinumt.

## RESULTS

### Discovery and frequency of NumtS across species

We identified a total of 409 polymorphic NumtS in 79 samples across 5 different non-human primate species (Additional file 1). Chimpanzee was observed to encompass the largest number of polymorphic Numt insertions with a total of 187 events discovered in 24 samples across four different subgroups at an observed rate of 20.8 average per sample. *Pan troglodytes troglodytes*, *P. troglodytes ellioti*, *Pan troglodytes schweinfurthii* exhibited an average of 22 events per sample, though *P. troglodytes*  *verus* showed a much lower rate of 10.2. Macaque had the highest overall average per sample rate of 21.2 insertions per sample and a total of 56 unique events called in five samples, while orang-utans showed the lowest at 12.9 average per sample, with 43 total unique events in 10 samples from two different species, though with differing rates between *Pongo*  *pygmaeus* (17.2 avg/sample) and *Pongo abelii* (8.6 avg/sample). The bonobo individuals in aggregate had 67 total insertions with an average of 15.46 per sample and 27 samples of gorilla had a total of 55 Numt called in two different species and three different subspecies with an overall 14.6 avg/sample. Both *Gorilla gorilla gorilla* and *Gorilla gorilla diehli* had approximately 15 events per sample, but the subspecies *Gorilla beringei graueri* only had 2.5 avg/sample, similar to human polymorphic events (Table 1). We reexamined the reference genomes for each species as many had been updated since previous studies on NumtS had been conducted and cataloged a total of 4274 reference (fixed) NumtS in great apes and macaque (chimpanzee: 974; bonobo: 809; gorilla: 657, orang-utan: 963 and macaque: 871) (Additional File 2).

**Table 1.** Frequency of NumtS

	Frequency of Numt insertions					
Species	N	Coverage	Reference	Polymorphic		Ave/sample
				Spp	Sub_Spp	
Homo sapiens*	999	4-12	767	141		1.5
***Pan*** ***troglodytes***	24	4.4	918	187		20.8
*Pan troglodytes troglodytes*	4	6.2			67	25.7
*Pan troglodytes ellioti*	9	3.5			98	21.8
*Pan troglodytes verus*	5	5.7			41	10.2
*Pan troglodytes schweinfurthii*	6	5.2			78	23.6
***Pan panicus***	13	5.9	736	67		15.4
***Gorilla***	27	6.4	657	55		13.6
*Gorilla gorilla gorilla*	24	6.8			48	14.4
*Gorilla gorilla diehli*	1	6.2			16	16
*Gorilla beringei graueri*	2	6.3			5	2.5
***Pongo***	10	6.4	923	44		12.9
*Pongo abelii*	5	6.1			34	8.6
*Pongo pygmaeus*	5	6.6			40	17.2
***Macaca mulata***	5	9	872	57		19.4
**Total**	1078		4873	432		

The frequency of Numt insertions in reference genomes and Numt insertions relative to the respective reference, discovered in several (*N* = number of samples) samples of six different groups (human, chimpanzee, bonobo, gorilla, orang-utan and macaque). Chimpanzee and gorilla are further divided into subspecies. The total polymorphic NumtS for the species are listed under ‘spp’ whereas the frequency for subspecies are listed under ‘sub_spp’. Average coverage for each group is noted in the third column. The last column has the average Numt insertions in each group. *Reported previously in (7)

**Figure 1.**
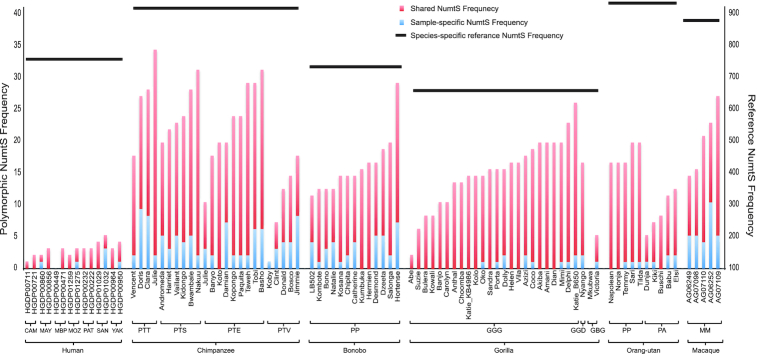
Shared and sample-specific Numt frequency. Each bar is representing the frequency of Numt insertion in that sample. The red portion of the bar indicates the number of events that are shared between samples within that species, whereas the blue bar shows the Numt insertions unique to that sample. Ass three-letter code is used for grouping the subspecies (in case of chimpanzee and gorilla) and population (for humans), whereas a two-letter code is used for denoting the species (for bonobo, orang-utan and macaque).

We next assessed the frequency of sample-specific NumtS and observed a common trend in chimpanzee, bonobo and macaque for a higher rate of sample-specific, relatively recent insertions compared to other species. In contrast, most insertions in gorilla and orang-utan were shared among other members of the species (Figure 1). We also discovered several events that were found in almost all samples suggesting they may represent older insertions. Overall, a higher frequency of polymorphic NumtS was observed in chimpanzee subspecies, with the exception of *P. troglodytes*  *verus* exhibiting a frequency similar to the other primates. Furthermore, we did not observe any effect of insert-size or coverage on the number of insertions (Additional File 3: Supplementary Figure S1).

### Spatial organization of Numt insertions into nuclear primate genomes

We examined the chromosomal locations of Numt integration across homologous regions of each species to explore shared insertional preferences. We found no indication of preferential bias toward specific chromosomes or regions for either NumtS present in the respective references of each species or polymorphic events (Additional File 3: Supplementary Figure S2A and B), with the exception of chromosome Y. No polymorphic insertions were detected even for species in which the Y chromosome was available for analysis (humans, chimpanzees and orang-utans). There were also several gaps on some chromosomes in various species that may be due to variability in assembly, particularly around the centromeric regions, potentially resulting in a dearth of Numt detection.

### *De novo* assembly of Numt insertions

**Figure 2.**
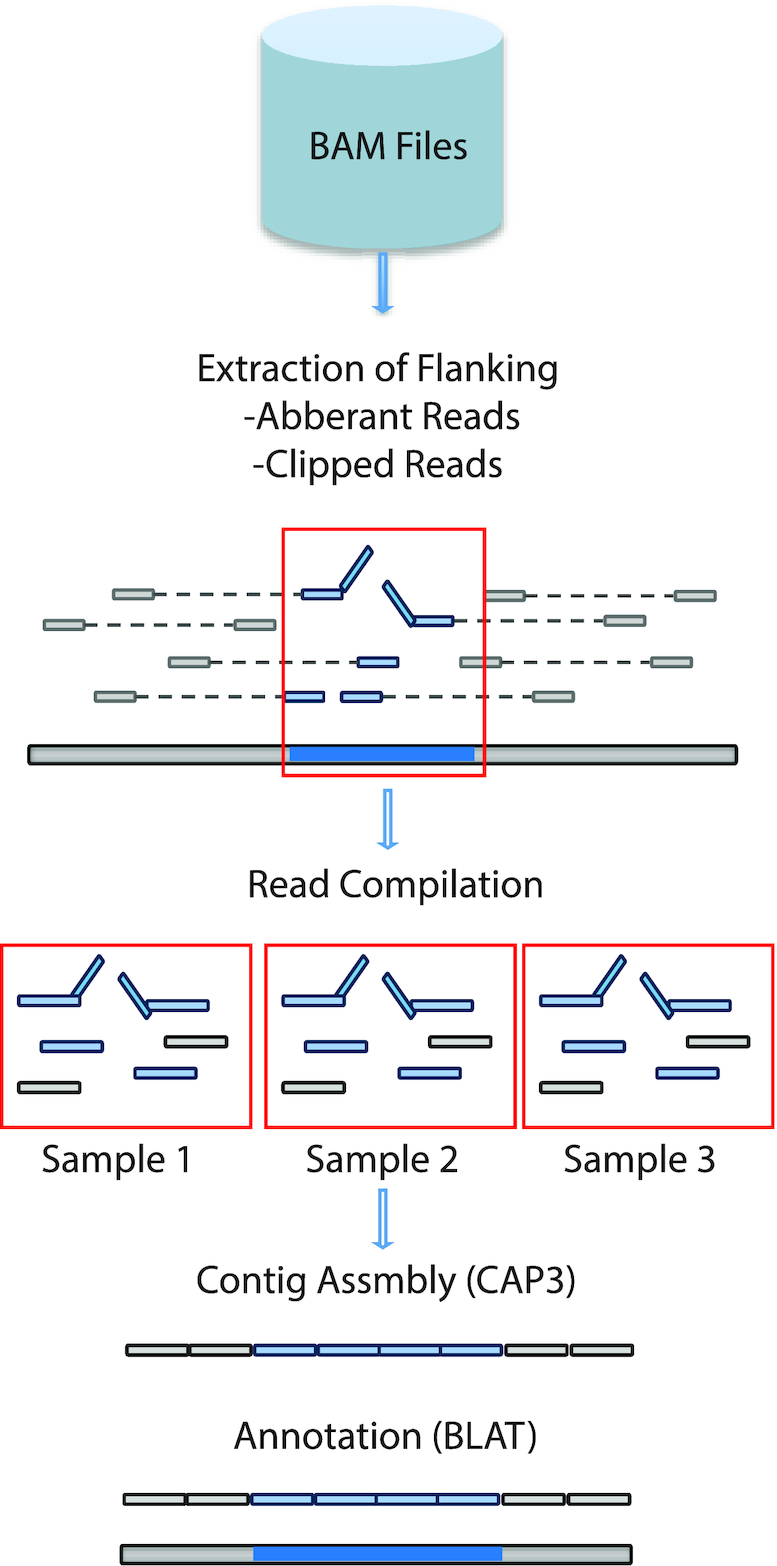
Pipeline for Numt assembly. The graphical representation is divided into three main parts of the Numt assembly pipeline. (**A**) Utilizing BAM files to extract the flanking reads and split reads around the breakpoints of Numt insertion from all the samples that they were discovered in. (**B**) These reads are then combined into one file to assemble into contigs using CAP3. (**C**) The contigs are then annotated using UCSC BLAT feature.

We next sought to derive the underlying sequences of the Numt insertions. In contrast to our earlier work with human cell lines, the underlying primate samples from which the whole genome sequences were derived were either unavailable or very sparse and thus prohibit the direct molecular characterization of these events. We thus applied an assembly strategy (see ‘Materials and Methods’ section and Figure 2) around each putative insertion site and were able to construct assembled contigs for 54% of our predictions across each species. We assessed the accuracy of our assembled sequences through comparison with high-quality NumtS we had previously sequenced and observed a very high accuracy at concordant sites (99.9% average identity). Using this approach, we were able to recapitulate the underlying insertion sequences for 222 NumtS across all species (Additional Files 4–8). The majority of NumtS that we were able to assemble were found in multiple samples. Longer/full-length NumtS were more difficult to assemble due to the circular nature of mtDNA as well as the limitations of using short reads, though we were able to derive the estimated length and mitochondrial region of origin for some longer insertions based on the partial assembly of each breakpoint.

### Validation of Numt calls using long-read sequencing

PacBio long-read sequencing can generate longer (10 Kbp or higher on average) contiguous DNA sequences than standard short-read sequencing technologies and can identify full-length insertions that fall completely within an individually sequenced molecule (30). We made use of three primate genomes (one chimpanzee, one gorilla and one orang-utan) sequenced to >65× by PacBio to provide orthogonal validation for our call sets of NumtS (28). Although these sequences were obtained from different individual samples and were not present in the short-read cohort we analyzed, we postulated that it would still be likely that we would identify some species-specific polymorphisms that were shared between the sets that could be used as a basis for comparison. Indeed, we observed orthogonal evidence for seven NumtS in chimpanzee, four NumtS in orang-utan and one in gorilla (Additional File 9) from PacBio data. The PacBio long reads also provided augmented information for these calls, including the specific sequence and segment coordinates within the mtDNA as well as insertion orientation (Additional File 9). In addition to verifying the insertion loci in the nuclear genome, we further compared our assembled NumtS to the sequences present in the respective PacBio long reads and found a 99.5% average identity concordance between the two technologies. Together, the results showed a high consistency between calls by PacBio data and our assembled Numt insertions by standard short-read technology with regard to insertion loci, segment coordinates within the mtDNA, and the insertion sequences suggesting we obtained a high confidence set of polymorphic assembled Numt insertions.

### Characterization and enrichment of NumtS

**Figure 3.**
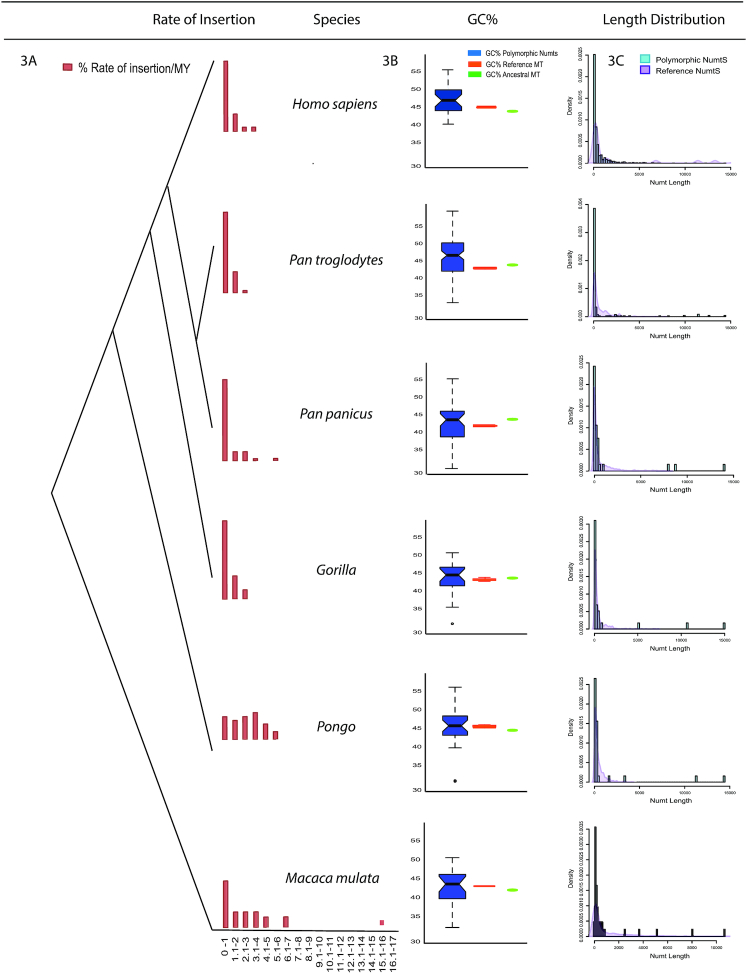
Rate of insertion, length of NumtS and GC content. (**A**) Rate of Numt insertion represented by the red bars divided per MYs for all 6 groups. (**B**) %GC content in assembled polymorphic Numts compared (represented by blue notched box plot) with %GC content of reference mtDNA (orange bar) and ancestral mtDNA (green bar). (**C**) Length of assembled polymorphic NumtS represented by the blue bars compared to the length of reference NumtS represented by overlain purple density plot.

We previously reported that polymorphic NumtS in humans exhibited a higher GC% than their parent mitochondrial genome (7), and thus we sought to determine whether this observation extended to non-human primates as well. We calculated the GC% for the subset of NumtS we were able to assemble for each species and, unlike humans, observed no significant change in any of the polymorphic insertions compared to their parent mtDNA with the exception of those present in chimpanzee (Figure 3). However, the GC% for all polymorphic events differed between species, with those within macaque, gorilla, and bonobo exhibiting a lower GC% in contrast to the higher GC% observed in human, chimpanzee and orang-utans (Additional File 3: Supplementary Figure S3). A similar observation was also noted in average GC% for reference mtDNA in all species. An increase in GC% of polymorphic events compared to their respective reference mtDNA was observed for chimpanzees and humans. This suggests that while polymorphic events mimic the GC% of their parent mtDNA in all other species, chimpanzees and humans might have a selective bias for higher GC% fragments for recently inserted polymorphic NumtS. We further generated a phylogenetic tree using the reference mtDNA for each species and their respective ancestral mtDNA to explore ancestral relationships and found species-specific mtDNA to cluster perfectly with their ancestral mtDNA (Additional file 3: Supplementary Figure S4). Additionally, we also found that the average GC% for macaque, orang-utan and human ancestral mtDNA was lower compared to GC% of polymorphic NumtS and reference mtDNA, whereas it was similar or higher for the rest of the species (Figure 3).

We next assessed whether there were length differences between our polymorphic NumtS and those present in their respective reference sequences (Figure 3). The majority (∼95%) of the assembled polymorphic insertions were smaller than 1 Kb in length, which is expected given the limitations of short-read assembly and is consistent with our previous results (7). Even though we were not able to fully assemble these long insertions, we were able to partially assemble contigs that crossed over the breakpoints precisely and matched our predicted mtDNA coordinates, enabling us to infer that ∼5% of the events were likely full length. In contrast, reference insertions across all species were up to 8 Kbp in size, with 3 (>8 Kbp) outliers only in humans.

To get an overview of fixed Numt evolution spanning over millions of years, we characterized the ancestral NumtS at each node of species divergence. A total of 530 shared events between human, chimpanzee and bonobo were observed, of which only two human polymorphic NumtS were shared with chimpanzee reference NumtS suggesting they may actually represent human-specific deletion polymorphisms. At the next node of divergence, we found a total of 426 events shared between human, chimpanzee, bonobo and gorilla, further decreasing to 355 where orang-utan diverged with the rest of primates. Finally, a total of 132 shared NumtS were characterized by the common ancestor between macaque and primates (Additional File 3: Supplementary Figure S5A). The human coordinates of all the 530 shared NumtS with each of the five other species, including the two polymorphic shared events (denoted by ‘HP’) can be found in Additional File 10. As expected, we observed the least number of shared NumtS at this node, given the 25MY’s of divergence. Due to the unusually low number of shared events between bonobo and the rest of the species, we decided to omit this group from further analysis to examine the shared events between groups. We observed the largest number of potentially fixed novel insertions occurring in chimpanzee and orang-utan (Additional File 3: Supplementary Figure S5B). Gorilla exhibited the fewest insertions, though this could also be due to its less-well characterized reference genome that still consists of several gaps. Finally, we conducted a pair-wise comparison between each species to look at the shared polymorphic and fixed NumtS. We observed that humans and chimpanzees have the most shared NumtS (Additional File 3: Supplementary Figure S5C). In contrast, bonobo showed a very low number of shared events with all other species, which was quite unexpected given the relatively short divergence period from chimpanzees. We also observed several additional shared polymorphic events between various species, suggesting either a deletion of reference events in the species or mischaracterization.

### Characterization of Numt insertional preferences in the nuclear genome

**Figure 4.**
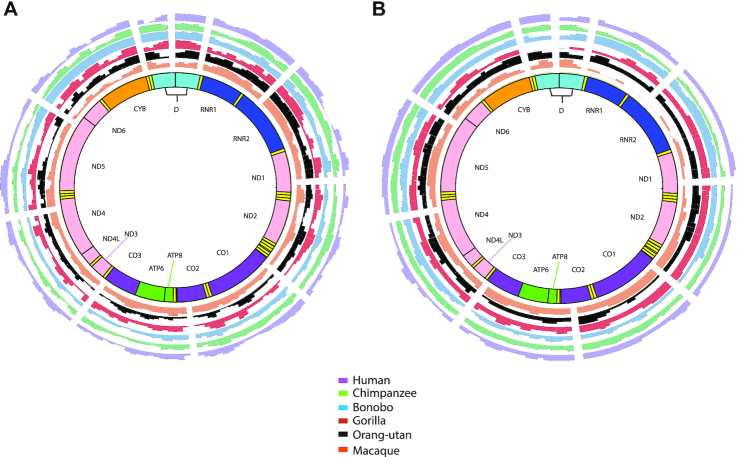
Spatial representation of species-specific insertions relative to the human reference genome. The breakpoints for all the species are lifted over to human reference (GRCh37/hg19). (**A**) The normalized frequency of each mitochondrial gene across all the reference Numt fragments. A unique color is assigned for each genus:purple for humans, green for chimpanzee, blue for bonobo, red for gorilla, black for orang-utan and orange for macaque. Using the same color scheme figure (**B**) was generated to illustrate the frequency of polymorphic NumtS.

**Figure 5.**
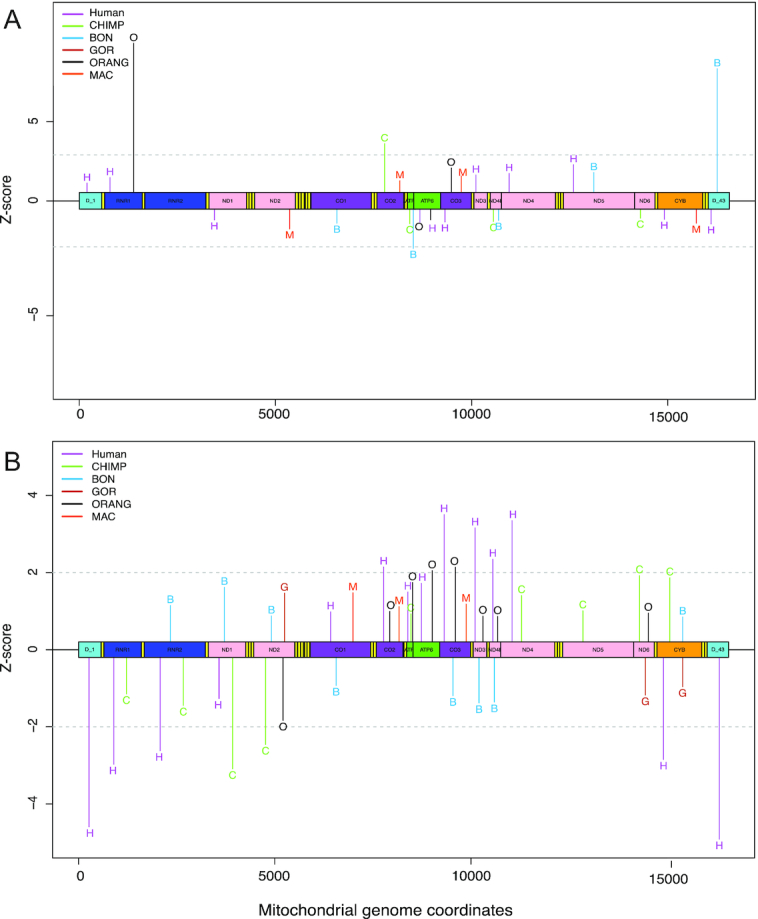
Mitochondrial gene enrichment or depletion across species. (**A**) Linear mtDNA representing the enriched and depleted (*Z*-score ≥ 1 and ≤ −1) major genes (genes coding for tRNA excluded) inserted as polymorphic NumtS in all six species. Unique color and alphabet is assigned for each genus, purple ‘H’ for humans, green ‘C’ for chimpanzee, blue ‘B’ for bonobo, red ‘G’ for gorilla, black ‘O’ for orang-utan and orange ‘M’ is for macaque. Using the same color scheme figure (**B**) was generated to illustrate the significant *Z*-scores for polymorphic NumtS.

We examined insertional preferences for any particular region of mtDNA, with a focus on Numt fragments containing complete genes or the D-loop (Figure 4A and B). We compared detected NumtS in the reference sequences of each species to random background models of insertions by converting gene annotations for mitochondrial genes for each species relative to human mtDNA using the liftOver tool (see ‘Materials and Methods’ section) and did not observe any obvious preferential bias toward any region or genes from the mtDNA relative to the human reference mtDNA sequence across all the species. We next examined polymorphic and reference NumtS identified individually at the species level and observed several regions of mtDNA that exhibited enrichment or depletion across multiple genes (Additional File 3: Supplementary Figures S6–11). We assessed these regions across all species to identify any selective biases toward particular MT genes and did not observe any pan-species MT gene bias for reference NumtS (‘Materials and Methods’ section, Figure 5A). However, for polymorphic events, we found an enrichment of genes located toward the center of mtDNA and depletion closer to the D-loop (Figure 5B). This was consistent across all species except bonobo, which interestingly exhibited the opposite, showing a depletion of genes toward the center of mtDNA and enrichment of D-loop containing fragments.

We next investigated whether there was any enrichment for polymorphic and reference NumtS within and across the different species The human genome (GRCh37) was segmented into bins of 25 Mbps to compare the frequency of Numt insertion (lifted over to the human reference) for each bin across all the groups (human, non-human primates, and *Macaca mulatta*) against a random background. Eight hotspots with a frequency greater than the threshold value (permutation threshold that defines 5% FDR) were observed for reference NumtS, one of which on chromosome 5 was also identified as the sole hotspot for polymorphic insertions (Additional file 3: Supplementary Figure S12). We further explored this region on chromosome 5 (chr5:75 000 000–100 000 000) to determine whether this was specific to an individual species or common across primates. We observed that the majority of these insertions were primarily from chimpanzee, bonobo and gorilla, while human, orang-utan and macaque were lacking any polymorphic insertions in this region. However, we did observe an overall high abundance of fixed reference events in all six species, with orang-utans exhibiting the largest number of insertions and humans the least. Additionally, to understand the evolutionary timeline of these insertions, we looked at how many of the shared (ancestral) events were a part of the hotspot on chromosome 5. We observed the same number (*n* = 6) of shared events between ‘humans, chimpanzees or bonobo’ and ‘humans, chimpanzees or bonobos and gorillas’. This decreased to four shared events when including orang-utan with these species. Only one ancestral shared event was observed in the hotspot region of chromosome 5 between all six species, implying this region began exhibiting multiple insertions after the divergence of macaque from the great apes.

Once a piece of mtDNA inserts itself into the nuclear genome, it typically follows the mutational rate of its host DNA that is an order of magnitude lower than the mtDNA, giving a snapshot of when the insertion occurred (1–3). Based on this, we calculated the rate of insertion per million years normalized against the evolutionary time of divergence. We observed that most polymorphic insertions occurred in the past million years for gorilla, bonobo, chimpanzee and macaque, which was similar to what has been observed for humans. orang-utan was the only group that showed a different rate of insertion, where the insertions were spread evenly over the past 4 to 5 million years (Figure 3). Interestingly, we also found a Numt that inserted ∼15 MYA in the macaque. It is possible however that these insertions are fixed in the population but are not a part of the reference due to weak characterization and gaps in some of these primate species.

## DISCUSSION

Advances in sequencing technologies have made it possible to conduct studies at a finer scale, allowing the exploration of previously understudied genomic variation in populations across multiple species. Here, we have described one of the first forays into the investigation of polymorphic nuclear mitochondrial insertions (NumtS) in multiple samples from primates and an old world monkey. We report a shift in the frequency of polymorphic Numt insertions with a successive increase from orang-utans to chimpanzees, followed by a decrease in humans by an order of magnitude. The highest rate of insertion was observed in *Macaca mulatta* which diverged from humans approximately 25 MYA. This suggests that at some point after macaque-primate divergence there was a considerable drop in the rate of insertion, as seen in orang-utans. Indeed, a burst of Numt insertions in the reference genome (and thus, primarily fixed in the species) has been previously reported (15) around the same period as the divergence of old world monkeys, which is consistent with their presence as an outgroup in our analysis. Although we can posit no specific cause for the change in the rates of insertions, we can proffer several speculations. First, this might be due to Mitochondria's rate of disintegration caused by factors such as oxidative phosphorylation (35) unique to each species. Alternatively, the prevalent mechanism for Numt insertions is understood to be implicated with the repair of double-strand breaks (DSB) (8), however, there may be other mechanisms yet to be discovered that are specific to a given species.

We also observed an absence of polymorphic Numt insertions on the Y chromosomes across all species. This is in part due to the bonobo, gorilla, and macaque reference genomes omitting the Y chromosome, however, we also did not observe any polymorphic insertions in orang-utan, chimpanzee or even humans whose references are more complete. This suggests some selective pressure against these insertions on Y chromosome; alternatively, if the insertions were to occur in the gamete stage when only the ovum contains mtDNA and not the sperm, the absence of any insertions on the Y chromosome might be explained. This hypothesis is further supported by the findings shown by (18) that all the recent reference NumtS on the Y chromosome were due to the result of duplication rather than novel insertions.

We observed a higher GC% of polymorphic NumtS in chimpanzee and humans compared to their respective parent mtDNA, which might be due to two reasons; i) If there is an insertional bias toward mtDNA fragments with higher GC%, to balance the low GC content of the region where the insertion occurs on nuDNA or ii) the mtDNA fragment with lower GC% disintegrating faster than the fragments that have a higher GC, thus increasing the chances of these fragments being selected for repairing the non-homologous breaks.

We further report an enrichment of both reference and polymorphic Numt insertions on chromosome 5. While for reference events the hotspot was derived by a substantial number of insertions across all the species (especially orang-utan with 50 insertions), polymorphic events were mainly derived by gorilla and bonobo. Combined with the low number of shared NumtS observed at each node of divergence, it suggests that these hotspots were created by insertions that are an ongoing and independent occurrence across all of the species. We did observe that human reference (GRCh37) has a slightly lower average GC% at 34% for 100 bp flanks on either side of Numt breakpoints in this region. Further, (36) examined structural variations and transposable elements by dividing the genome into 10 Kbp bins and reported several hotspots in this 25 Mb region for human (9), chimpanzee (6) and orang-utan (6). Interestingly, they did not find any hotspots in rhesus. So far the only known mechanism for Numt insertions is through the repair of DSBs, but exploring this phenomenon could potentially lead to other mechanistic explanations for Numt insertions and their continued occurrence. It should be noted that the above mentioned observations might also possibly be because some of the primate references are not well defined and we might be missing either newer NumtS relative to the reference or calling events that are fixed in the species but missing in the reference. We also note that there is likely an ascertainment bias however due to the preferential assembly of smaller NumtS events from the short-read sequence data available.

Our study does have some limitations, such as the lack of direct molecular validation due to the unavailability of the samples we analyzed. However, we previously established the high fidelity of our NumtS detection method in humans (7) and expect a similar accuracy for the non-human primates assessed here. We also made use of the long-read PacBio sequences which, while limited, had very good concordance with both our predicted insertions as well as their assembled sequences. This indicates that our *de novo* assembly approach can serve as a valuable tool for when exploring such insertions in nuclear genomes for datasets where the further molecular characterization of samples is not possible. We also acknowledge the potential bias created due to poorly assembled references or gaps for certain species. However, by characterizing shared events between species, we have been able to derive the most comprehensive overview till date of the evolution of these NumtS across primates and macaque spanning over several million years of divergence.

## Supplementary Material

lqaa089_Supplemental_Files
