# Supplementary material for: Characterization of nuclear mitochondrial insertions in the whole genomes of primates
Source: NAR Genom Bioinform. 2020 Nov 16;2(4):lqaa089. doi: 10.1093/nargab/lqaa089 (PMC7671390; doi:10.1093/nargab/lqaa089)
Supplement: lqaa089_Supplemental_Files [file lqaa089_supplemental_files.zip › Additional file 3_Supplemental Figures.pdf]

Characterization of nuclear mitochondrial insertions in the whole genomes of primates

Gargi Dayama[1], Weichen Zhou[1], Javier Prado[3], Tomas Marques-Bonet[3], and Ryan E. Mills[1, 2*]

[1]Department of Computational Medicine & Bioinformatics and [2]Department of Human Genetics, University of Michigan Medical School, Ann Arbor, MI 48109

[3]Institut Biologia Evolutiva, Universitat Pompeu Fabra/CSIC, Aiguader 88, Barcelona, 08003

* To whom correspondence should be addressed. Tel: +1.734.647.9628; Email: remills@umich.edu

Supplemental Figures:

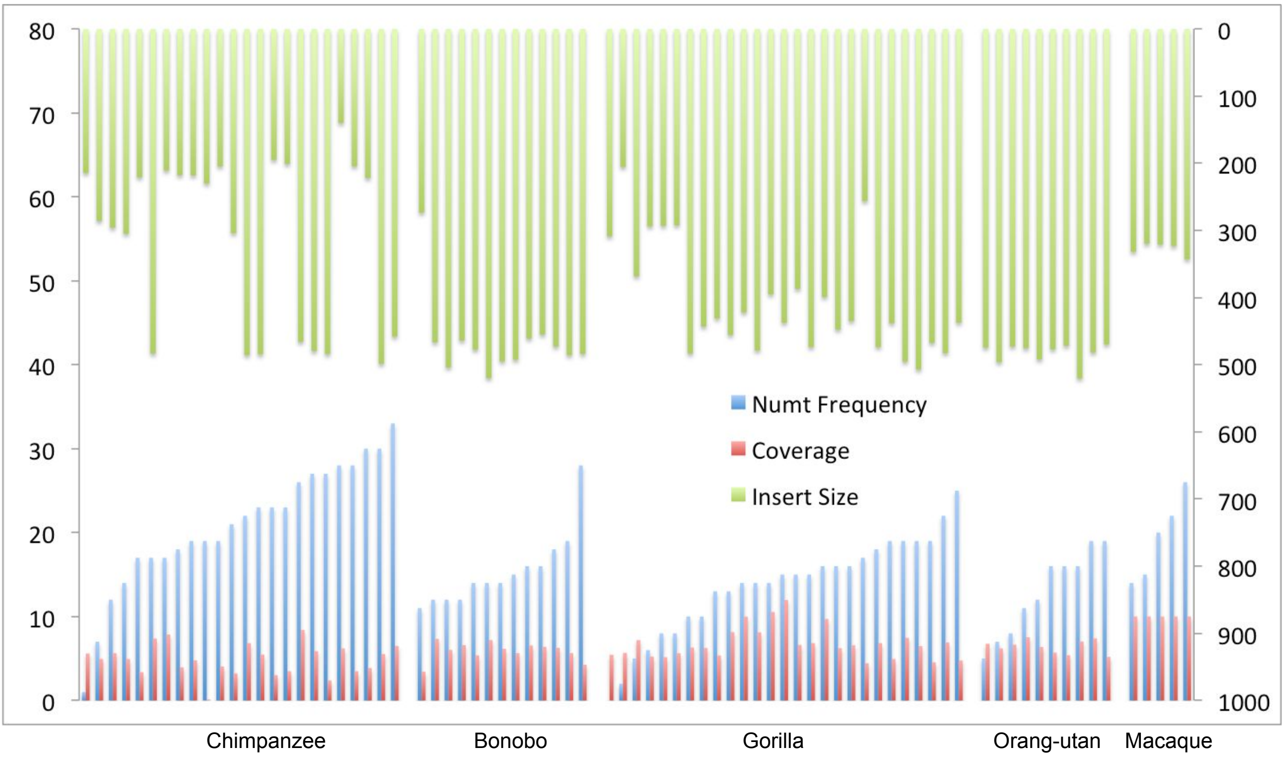

Fig S1. Numt frequency. The frequency of polymorphic Numt insertions (blue bars) relative to the sample coverage (red bars) and average insert size in bp (green bars) discovered in samples of 5 different groups (Chimpanzee, Bonobo, Gorilla, Orang-utan and Macaque).

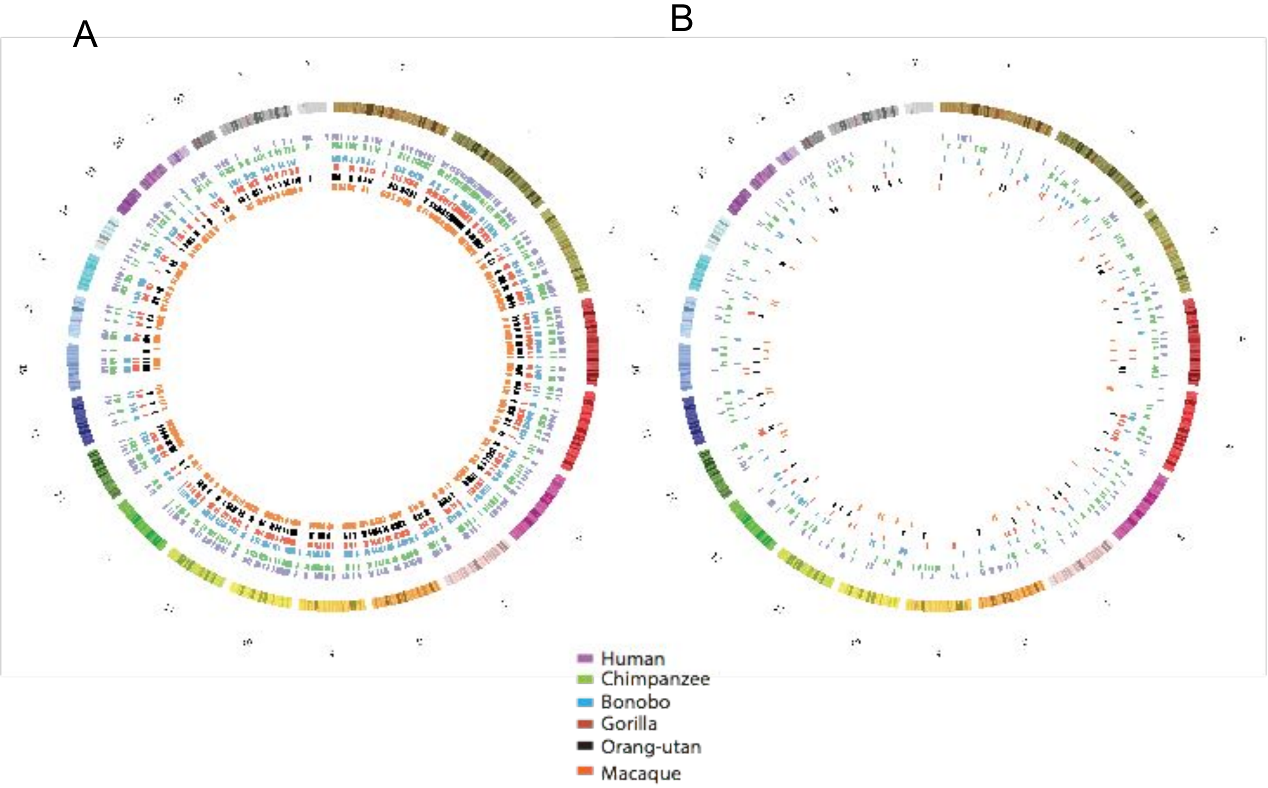

Fig S2. Spatial representation of species-specific insertions relative to the human reference genome. The breakpoints for all the species are lifted over to human reference (GRCh37/hg19). A) Position of Numt insertions for reference events. Unique color is assigned for each genus, purple for humans, green for chimpanzee, blue for bonobo, red for gorilla, black for orang-utan and orange is for macaque. Using the same color scheme figure B were generated to illustrate the Numt insertions for polymorphic events.

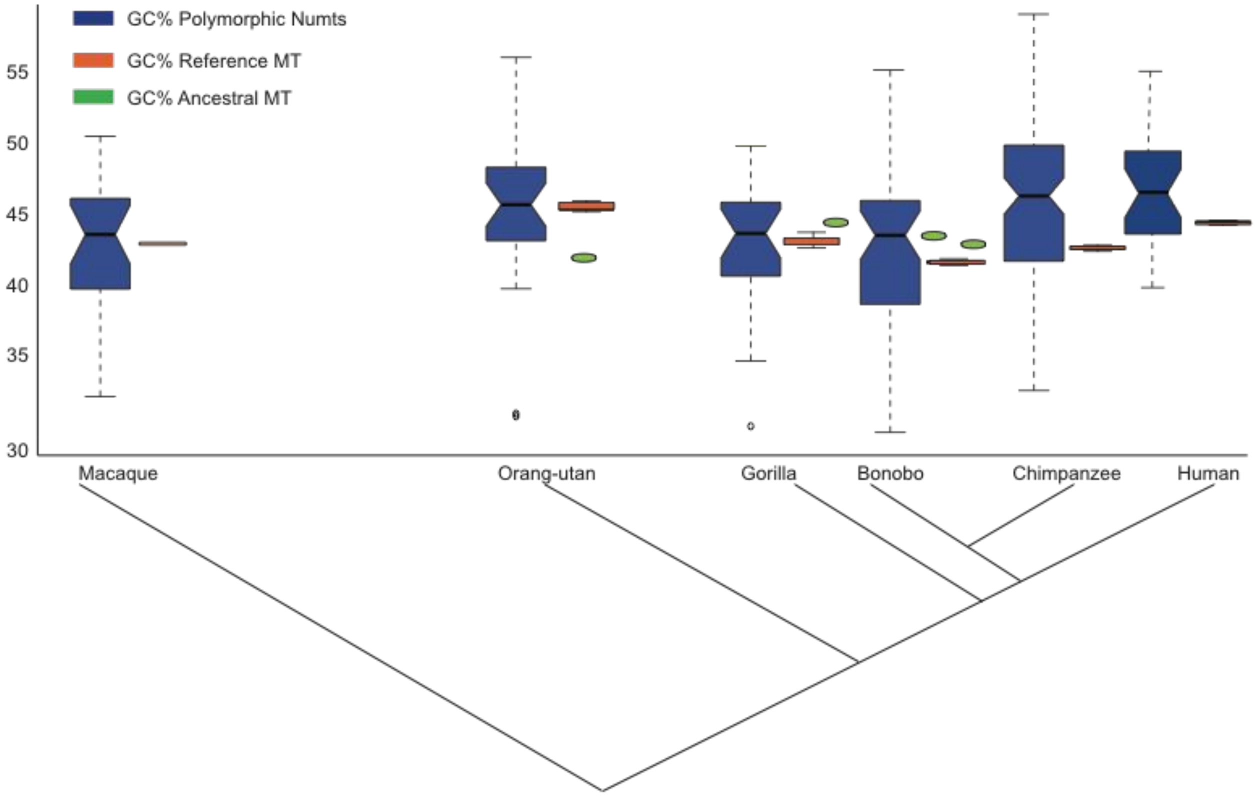

Fig S3. %GC for polymorphic NumtS, reference mtDNA and ancestral mtDNA. Comparing the GC% of polymorphic NumtS (blue) from each species to the average GC% of their respective reference mtDNA (orange) and respective ancestral mtDNA (green).

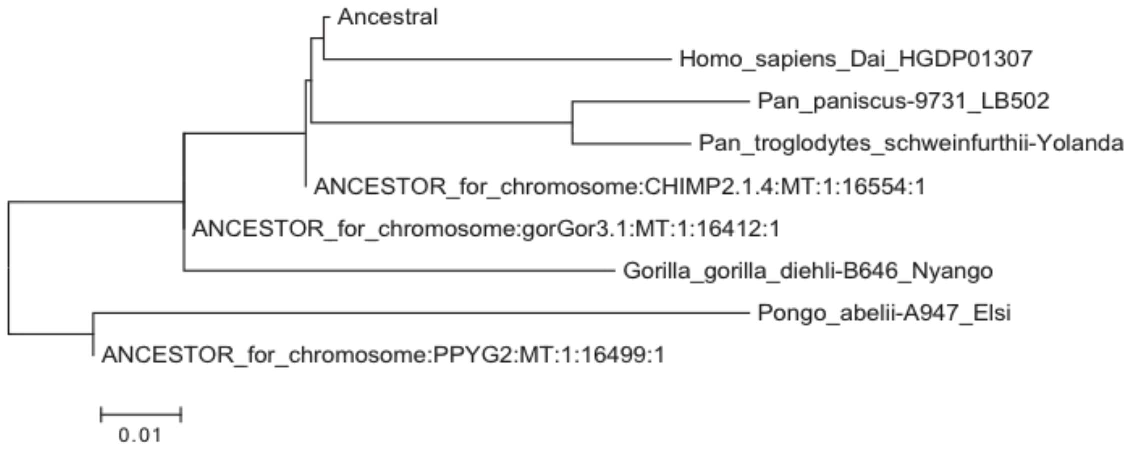

Fig S4. Phylogenetic tree. Comparison of ancestral and reference mtDNA to validate the link at each node of speciation.

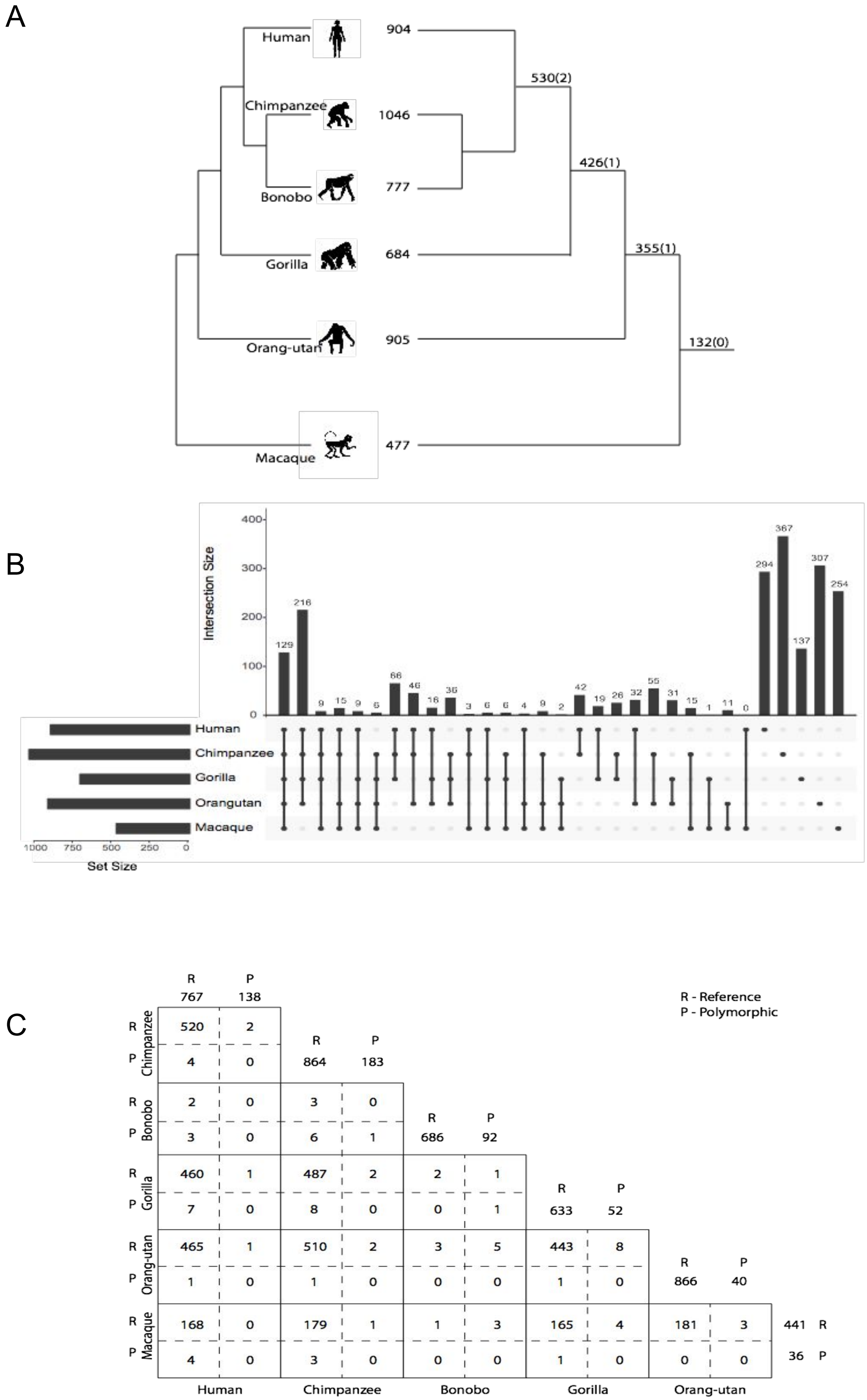

Fig S5. Shared and ancestral NumtS. (A) Intersecting fixed and polymorphic NumtS between the six different species to obtain ancestral NumtS (at each node). Human polymorphic NumtS shared with other 5 species reference events is represented in parenthesis. (B) UpSet plot representing the shared events between each combination of 5 different species. Bonobo was omitted to avoid bias because of its unusually low shared events (as evident in C). (C) Pair-wise comparison of fixed and polymorphic events between all six species.

A

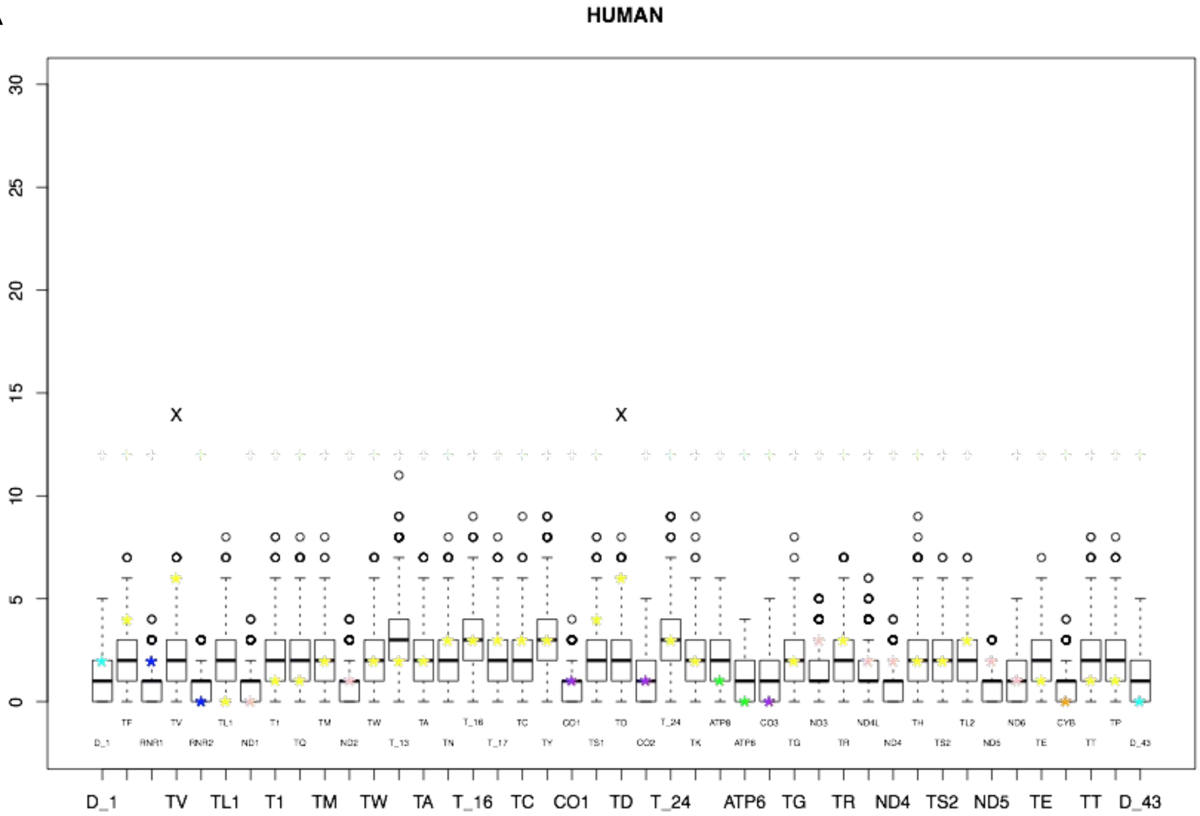

B

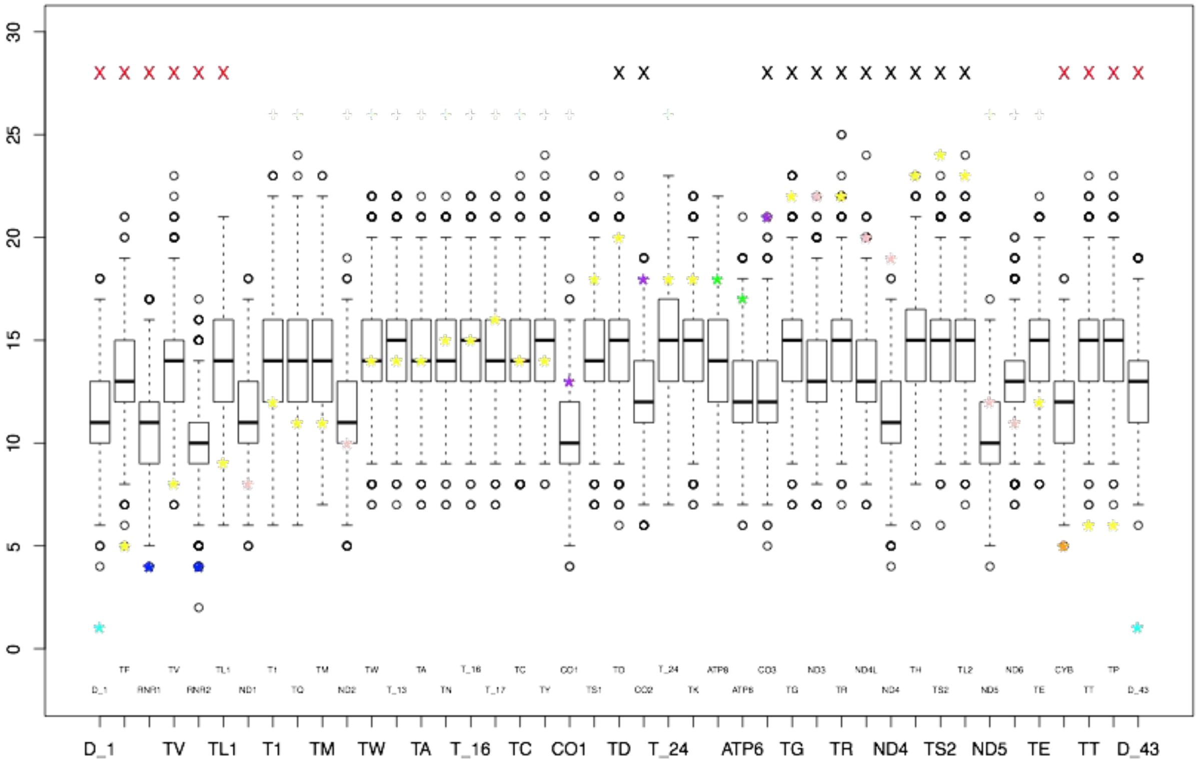

Fig S6. Enrichment analysis for complete mitochondrial genes integrated into the human genome, relative to human mtDNA sequence. Observations are indicated by asterisks and are relative to 1000 permutations of random mtDNA fragments of the same size (box plots). Significantly enriched or depleted genes (p-value <= 0.05) are denoted with a black or red X, respectively. Results are shown for (A) reference-based and (B) polymorphic NumtS.

A

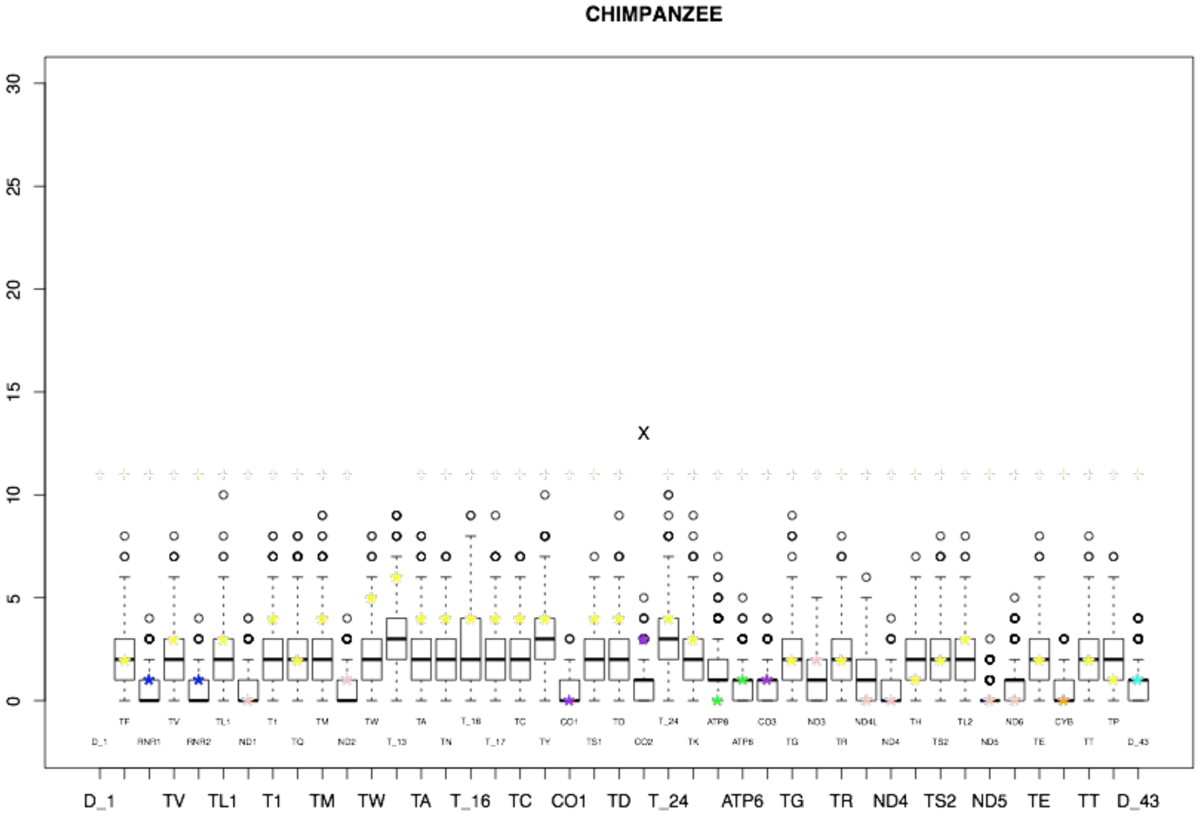

B

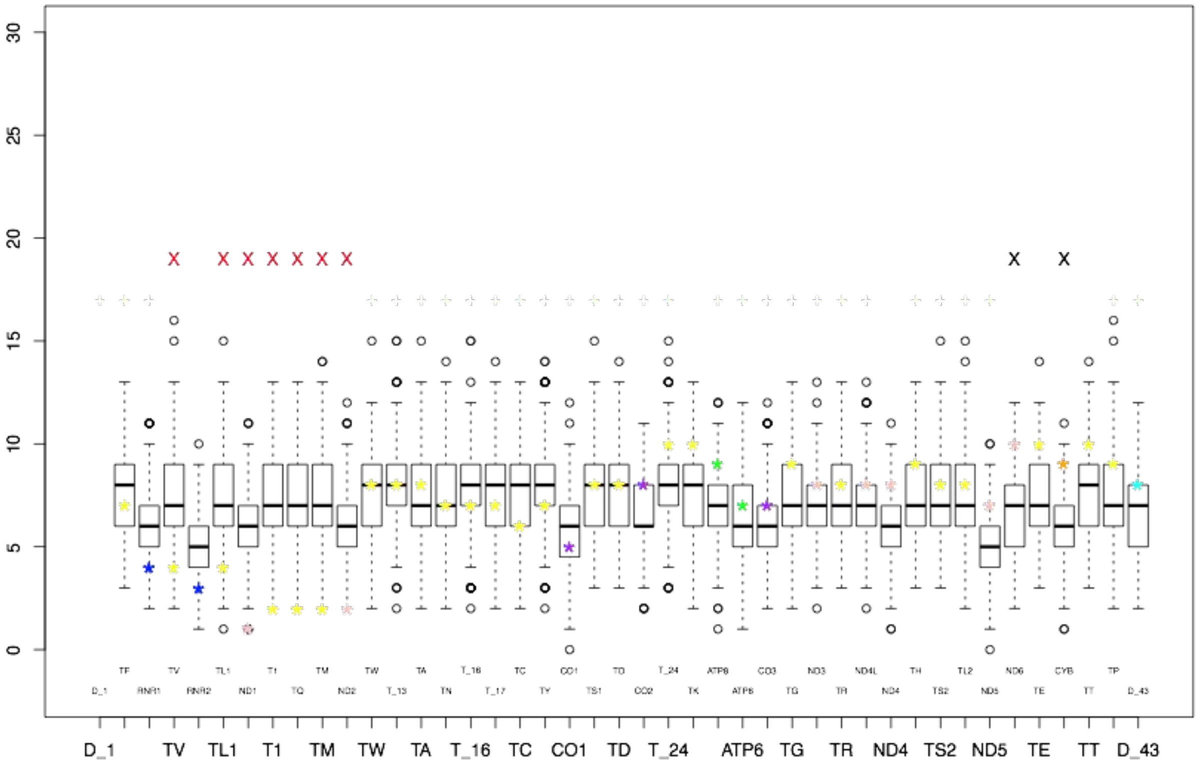

Fig S7. Enrichment analysis for complete mitochondrial genes integrated into the chimpanzee genome, relative to human mtDNA sequence. Data represented as described in Fig S3 for (A) reference-based and (B) polymorphic NumtS.

A

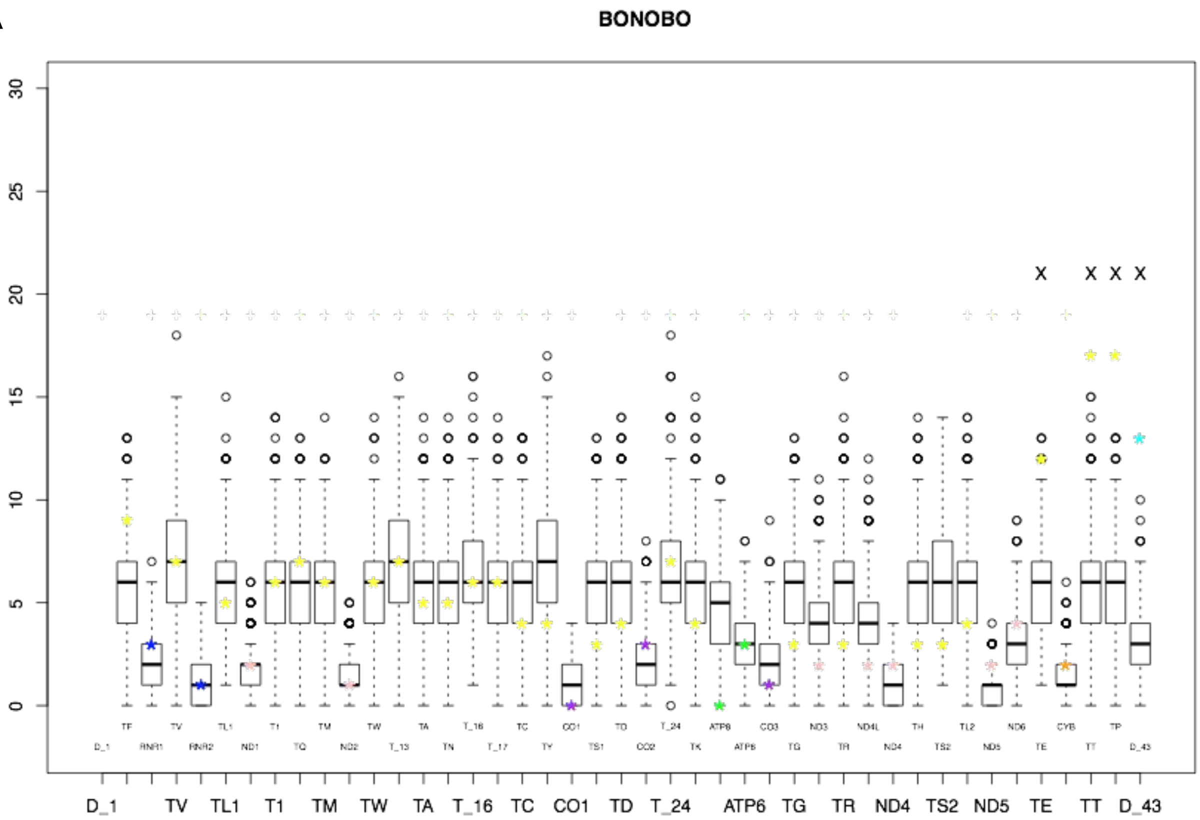

B

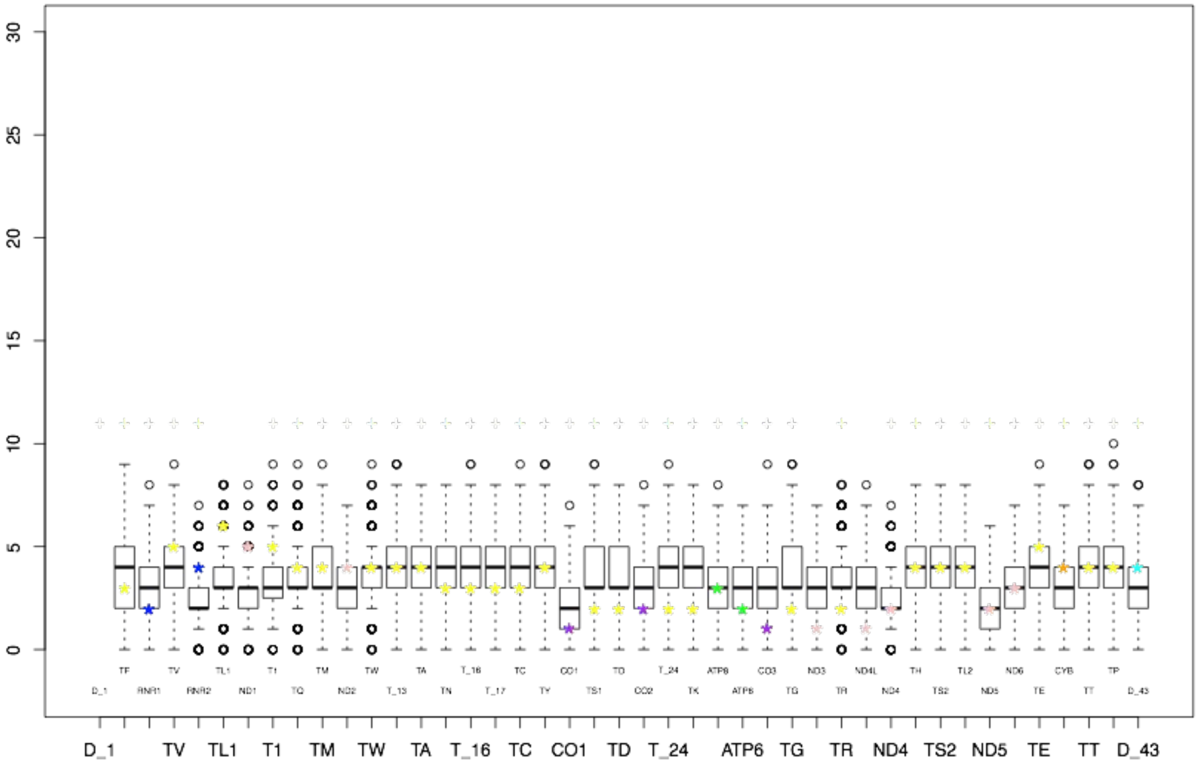

Fig S8. Enrichment analysis for complete mitochondrial genes integrated into the bonobo genome, relative to human mtDNA sequence. Data represented as described in Fig S3 for (A) reference-based and (B) polymorphic NumtS.

A

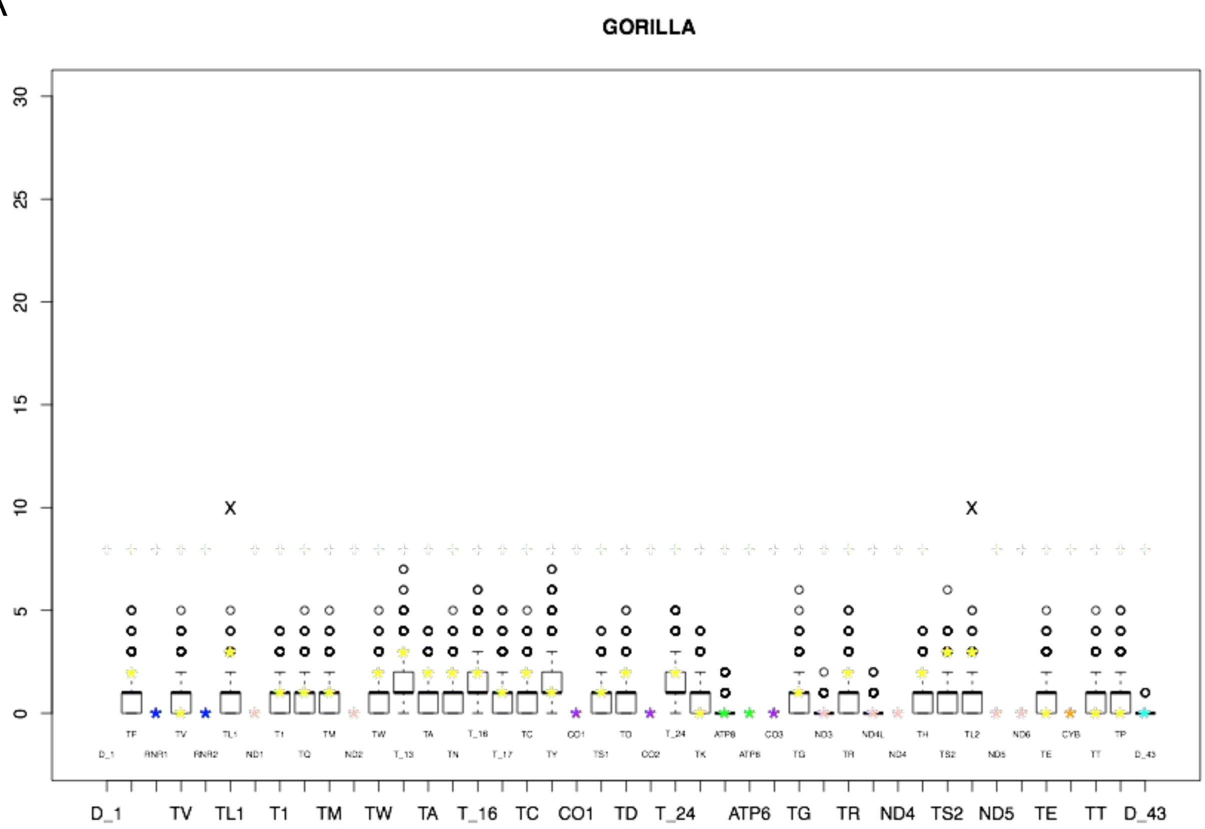

B

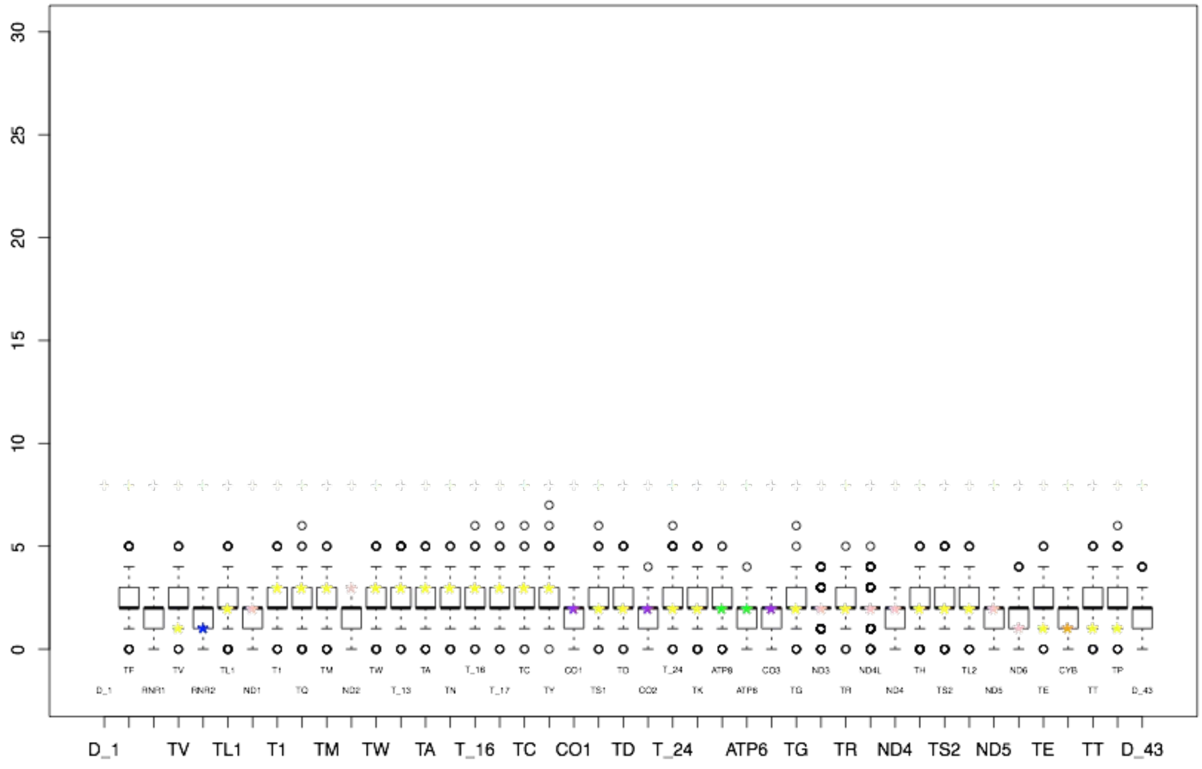

Fig S9. Enrichment analysis for complete mitochondrial genes integrated into the gorilla genome, relative to human mtDNA sequence. Data represented as described in Fig S3 for (A) reference-based and (B) polymorphic NumtS.

A

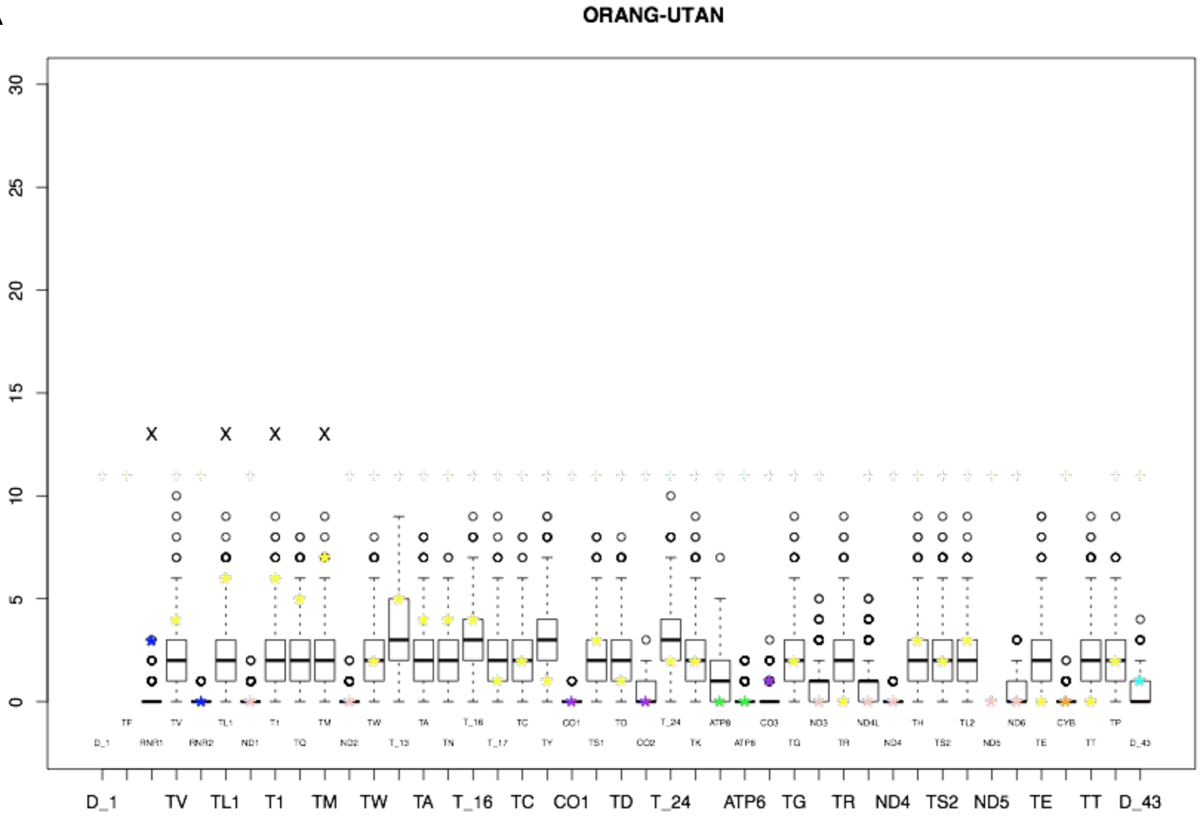

B

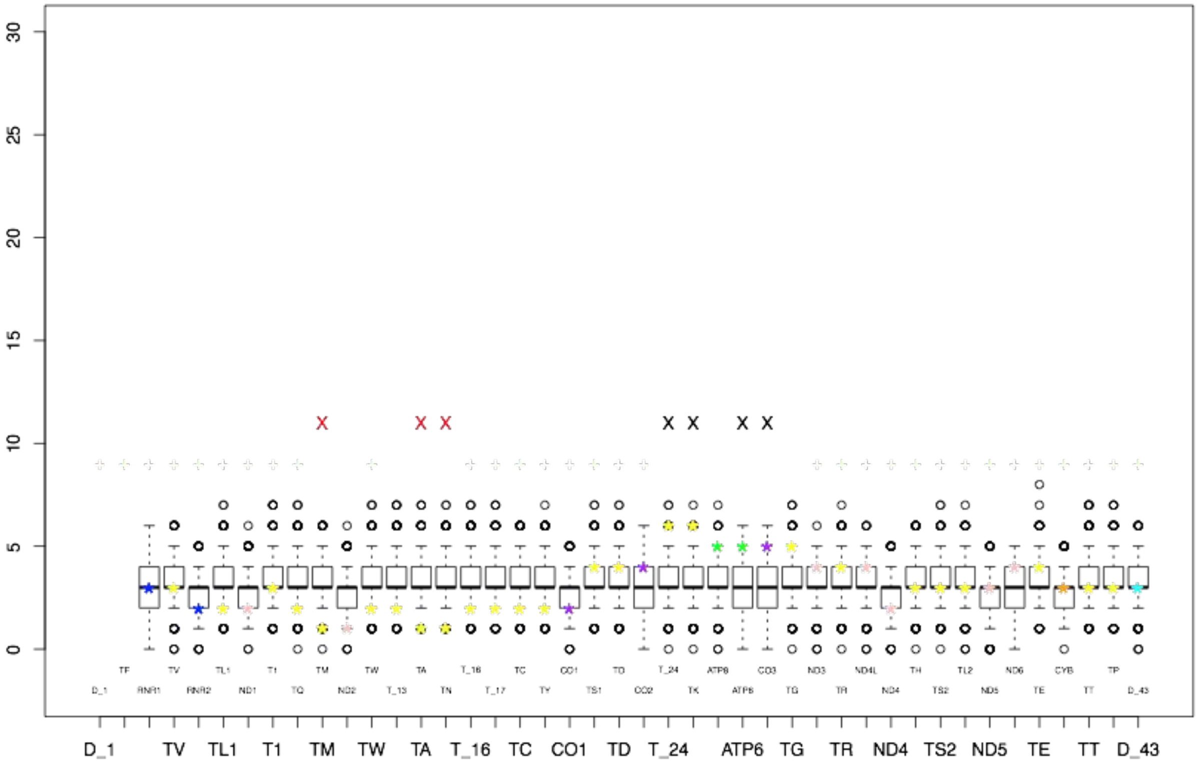

Fig S10. Enrichment analysis for complete mitochondrial genes integrated into the orang-utan genome, relative to human mtDNA sequence. Data represented as described in Fig S3 for (A) reference-based and (B) polymorphic NumtS.

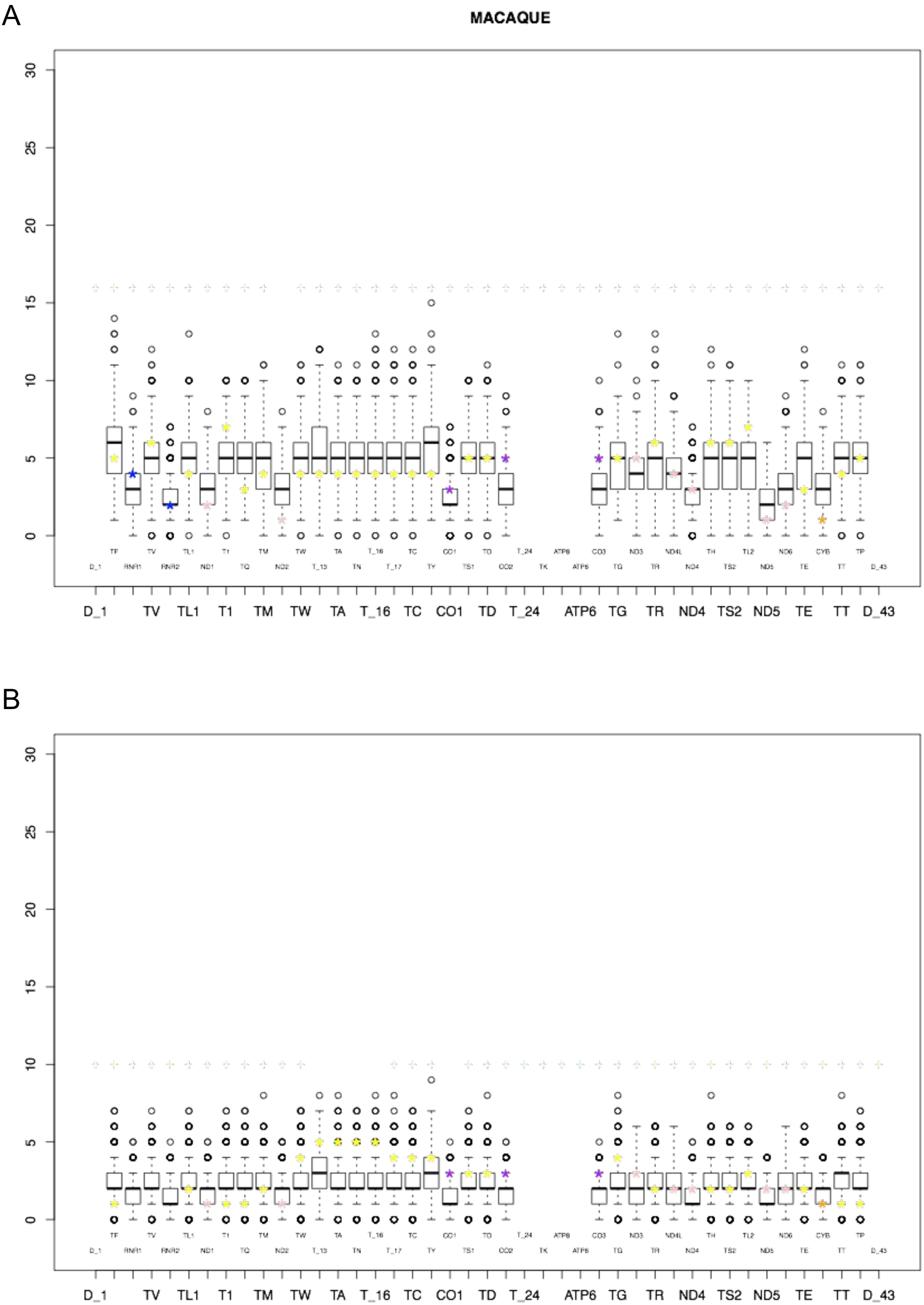

Fig S11. Enrichment analysis for complete mitochondrial genes integrated into the macaque genome, relative to human mtDNA sequence. Data represented as described in Fig S3 for (A) reference-based and (B) polymorphic NumtS.

A

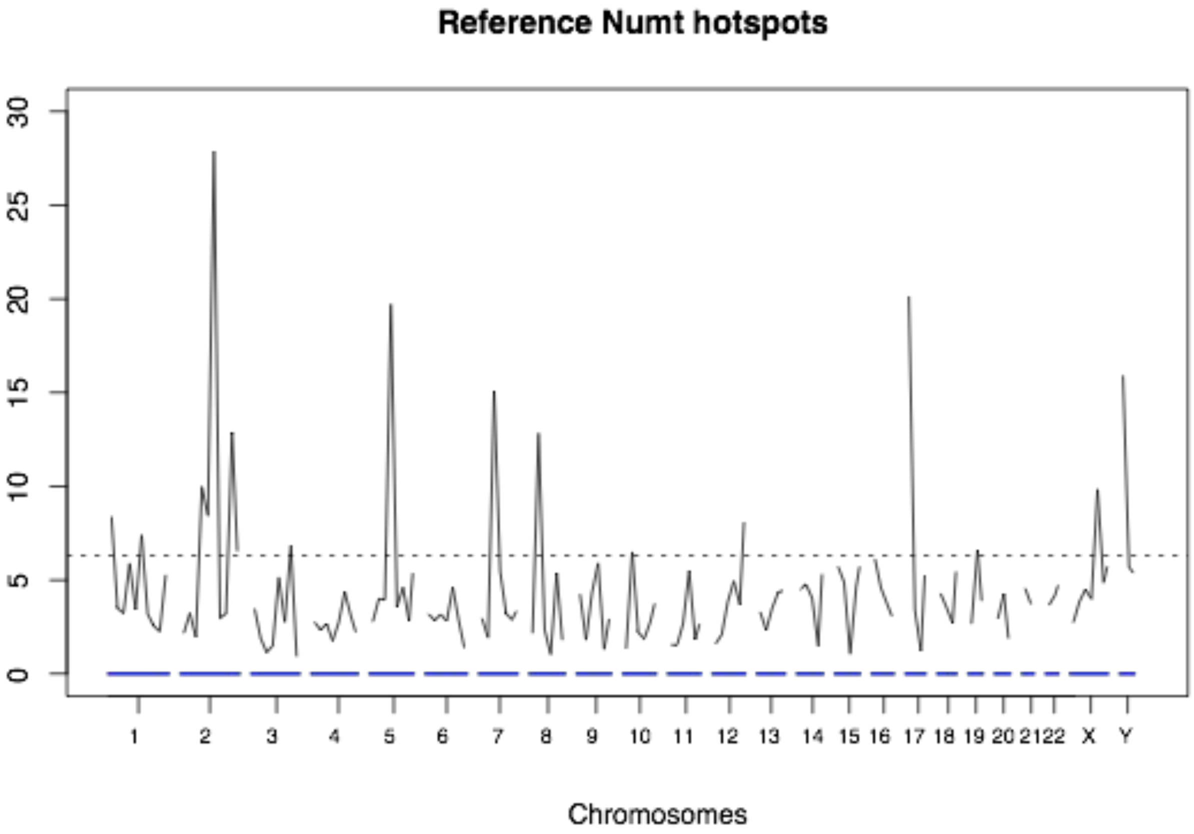

B

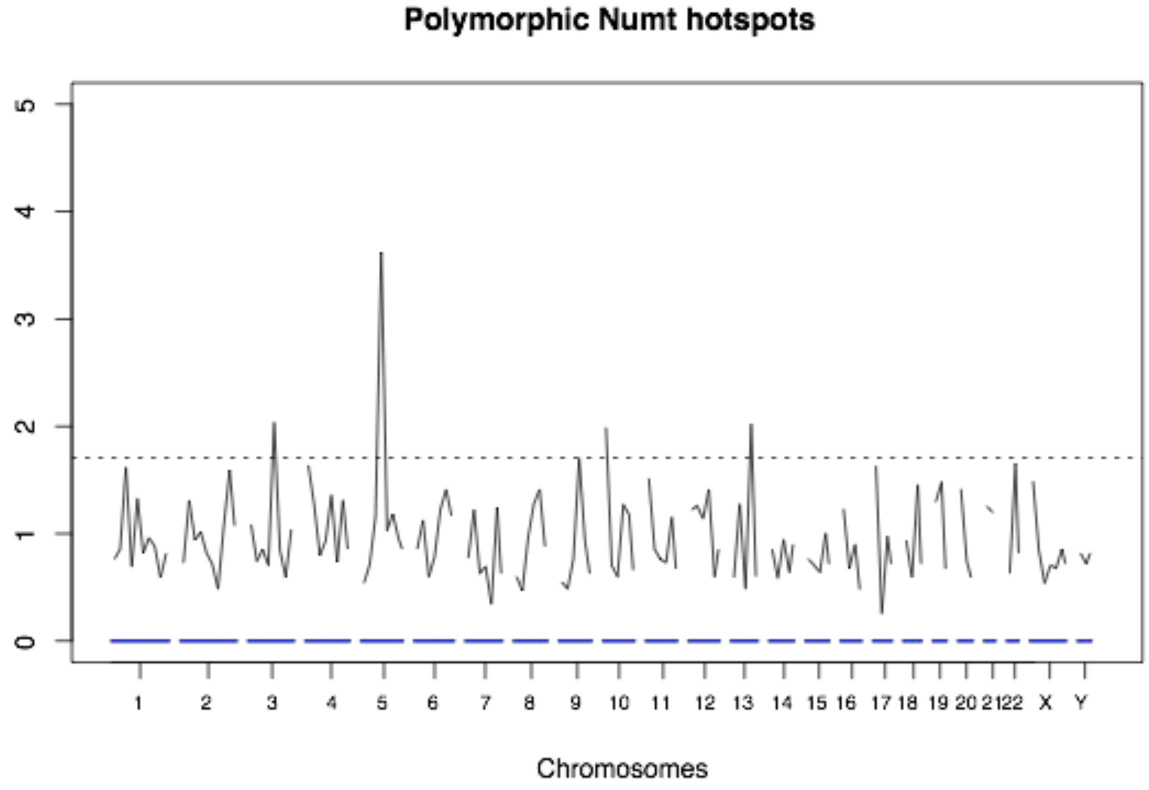

Fig S12. Hotspot analysis. Frequency of A) reference and B) polymorphic Numt events along the genome, compared across the homologous positions of the 6 species (see methods for hotspot frequency calculation). Each chromosome are divided into 25mbp bins. The dotted line is permutation threshold that defines 5% FDR (false discovery rate) of significant hotspots.
